# In silico study on the Hepatitis E virus RNA Helicase and its inhibition by silvestrol, rocaglamide and other flavagline compounds

**DOI:** 10.1038/s41598-022-19818-w

**Published:** 2022-09-15

**Authors:** Lorenzo Pedroni, Luca Dellafiora, Maria Olga Varrà, Gianni Galaverna, Sergio Ghidini

**Affiliations:** Department of Food and Drug, University of Parma, 43124 Parma, Italy

**Keywords:** Enzymes, Proteins, Biochemistry, Biological techniques, Bioinformatics, Biological models

## Abstract

Hepatitis E Virus (HEV) follows waterborne or zoonotic/foodborne transmission. Genotype 3 HEV infections are worldwide spread, especially in swine populations, representing an emerging threat for human health, both for farm workers and pork meat consumers. Unfortunately, HEV in vitro culture and analysis are still difficult, resulting in a poor understanding of its biology and hampering the implementation of counteracting strategies. Indeed, HEV encodes for only one non-structural multifunctional and multidomain protein (ORF1), which might be a good candidate for anti-HEV drugging strategies. In this context, an in silico molecular modelling approach that consisted in homology modelling to derive the 3D model target, docking study to simulate the binding event, and molecular dynamics to check complex stability over time was used. This workflow succeeded to describe ORF1 RNA Helicase domain from a molecular standpoint allowing the identification of potential inhibitory compounds among natural plant-based flavagline-related molecules such as silvestrol, rocaglamide and derivatives thereof. In the context of scouting potential anti-viral compounds and relying on the outcomes presented, further dedicated investigations on silvestrol, rocaglamide and a promising oxidized derivative have been suggested. For the sake of data reproducibility, the 3D model of HEV RNA Helicase has been made publicly available.

## Introduction

Hepatitis E virus (HEV) is a small positive sense single stranded RNA virus member of the *Hepiviridae* family (*Orthohepevirus* genus). It is the causative agent of the infamous Hepatitis E. As reported by the World Health Organization^1^, there are 20 million HEV estimated infections per year and 3.3 million symptomatic cases of Hepatitis E worldwide. The fatality rate is usually relatively low, ranging from 0.2 to 4%, although it significantly increases for pregnant women^2^.

The virus spreads following several routes with differences between high- and low-income countries. In the formers it is strictly related to zoonotic and foodborne transmission^3,4^ while in the latter it commonly gives waterborne outbreaks^5^. There are eight genotypes described so far (HEV-1 to -8), differing for host preferences and ways of transmission. Particularly, HEV-3, which is globally spread, and HEV-4, which is mostly limited to Asia, follow zoonotic and foodborne transmission^6,7^. Reservoirs of HEV include deer, wild boars, cows, sheep, and goats with evidence of human infection reported to be caused by contaminated milk^8–11^. Despite the wide range of potential hosts, the main reservoirs are pigs, and the related meat-based products are a major source of infection^12–14^. HEV was detected also in the berry fruit and leafy green vegetables supply chain because of irrigation with contaminated water^15,16^.

**Figure 1:**
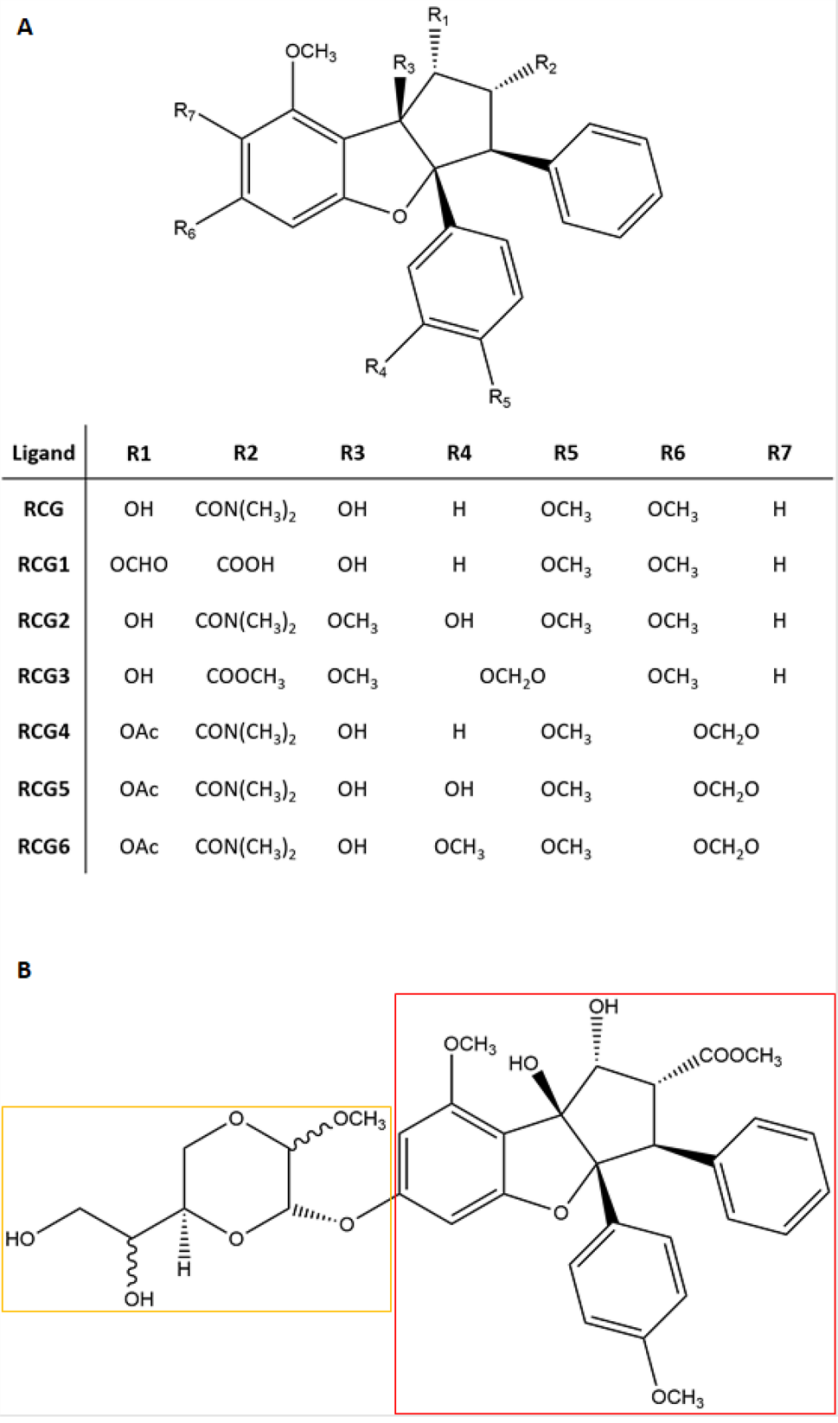
Chemical structure of RCG, RCG analogues and SLV. (**A**) RCG scaffold with its chemical substituents ranging from R1 to R7. (**B**) SLV chemical structure. The red square surrounds the RCG scaffold while the yellow the typical SLV dioxane portion. The latter is replaced by a hydroxyl group (–OH) in S–Ag.

Although this virus is widespread, in vitro culture and analysis are still difficult and consequently its molecular biology has not been fully understood yet^17^. One of the critical knowledge gaps concerns the HEV ORF1 gene, which encodes for a 185 kDa polyprotein with no cleavage sites reported^18^. ORF 1 is a multifunctional, multi-domain and non-structural polyprotein with a crucial role in the viral diffusion and replication, which may be a possible druggable target to interfere with the viral mechanisms of infection^19^. However, its huge dimension and multi-domain architecture make its analysis challenging via canonical molecular biology and structural approaches. In addition, the lack of available crystallographic and NMR structures hampers the identification and characterization of its druggable sites using canonical medicinal chemistry approaches. This lack of information ultimately prevents the structure-based identification of molecules targeting ORF1 domains for their possible implementation in anti-HEV strategies. In this context, in silico approaches can efficiently overcome the lack of structural data either to analyze proteins mechanics and druggability, or to provide a useful tool to study chemical and biological aspects of small molecules, including antiviral compounds^20–23^. For this reason, an in silico procedure has been developed and applied to target HEV ORF1. Based on previous studies showing the inhibitory activity of the natural plant secondary metabolite silvestrol (SLV; Fig. 1) against HEV-3^24–26^, our study provided a reliable model to: I) investigate the underpinning mechanisms and viral target; and II) to estimate the activity of SLV analogues for further dedicated investigations. To do so, the HEV RNA Helicase domain was modelled and refined via a homology modelling approach based on an innovative, hybrid structure- and sequence-based big-data analysis targeting the whole set of crystallographic data available so far in the Protein Data Bank (nearly 190.000 structures; last database access 28th February 2022). Then, the interaction of the model with the already known RNA Helicase inhibitor rocaglamide (RCG) and a set of 9 natural-related compounds^27^ was calculated through docking and molecular dynamics (MD) simulations.

## Results and discussion

### Building and refining the HEV RNA Helicase model

The HEV RNA Helicase domain of the HEV ORF1 polyprotein was meant to be modelled via homology modelling (HM), as reported in the "Homology modelling" section, due to the absence of a crystallographic structure in the Protein Data Bank. This method allows the successful modelling of proteins whose 3D structure is missing and it is particularly useful when crystallographic investigations are challenging, as in the case of polyproteins^28^.

Specifically, the HEV ORF1 domain’s coding sequence of HEV RNA Helicase was chosen based on the results reported by Karpe and Lole^29^ who succeeded to recombinantly express the putative HEV1 ORF1 RNA Helicase region (from amino acid 960 to 1204) proving its activity. The focus on HEV3 (UniProt AC Q6J8G2) was due to its prevalent foodborne/zoonotic transmission and its worldwide spread^30^. The localization of the RNA Helicase domain on the HEV3 ORF1 was achieved based on the alignment with the HEV1 RNA Helicase sequence proved as active by Karpe and Lole. Particularly, the target sequence (Tar-Seq) HEV3 ORF1 region ranging from amino acid 975–1219 shared 90.6% identity and 95.5% similarity with the HEV1 RNA Helicase domain (Fig. S3, Supplementary materials).

Concerning the template selection for HM, it is typically obtained identifying homologous sequences with available 3D structure via the BLAST UniProt web-interface, which is a gold benchmark standard in this kind of studies^31^. However, using the Tar-Seq as input for a canonical BLAST search gave no results. This was likely due to the nature of the BLAST heuristic algorithm that are likely to fail aligning polyprotein domains over their entire sequence. Indeed, the BLAST UniProt web-interface searches target sequences within the whole primary protein sequences associated with 3D data and not limiting the search to the actual sequence resolved in the crystal structure. However, in most of the medium/large proteins, such as polyproteins, the crystals related to the UniProt ACs partially cover the protein primary structure causing BLAST to fail in identifying homologs with known structures. To overcome this intrinsic weakness, a successful workflow was setup (see in "Sequence and template selection" section for further details). The modification in the search space used in this approach made it robust, reusable, and successful to find a useful template to build a model for a polyprotein domain. In more detail, all the entries having a 3D structure belonging to the Prokaryotic reign with E.C 3.6.4.13 (RNA Helicase activity, according to Brenda classification^32^) were downloaded from PDB. Then, 3D structure files were converted to FASTA files via an in-house script setting up a database which included the “crystallome” of the prokaryotic RNA Helicase available at the time of analysis. This database was finally searched for Tar-Seq homologous to identify a proper template for HM.

The best aligning protein among the 1940 PDB entries annotated as prokaryotic RNA Helicases at the time of analysis was chosen as template (see in the “Homology modelling” section for further details). It was the PDB entry having code 3WRY^33^, which showed identity and similarity percentage to HEV RNA Helicase of 24.5% and 39.2%, respectively, and with alignment score and e-value of 63.5 bits and 1e-12, respectively. Of note, the identity percentage was high enough with respect to the alignment length to ensure a reliable modeling procedure in agreement with previous evidence^34^. Such protein belongs to the *Tomato mosaic virus* (Tmv), a positive sense single stranded RNA virus belonging to the same HEV class. Before using it as template to model the HEV RNA Helicase domain, the last 160 N-terminal residues, which were organized in a self-standing sub-domain, were removed being not covered by our Tar-Seq. The model and its refinement process, reported in the "Model generation and refinement" section, allowed to obtain a reliable and stable model having 90% of the residues within the mostly favored regions, 10% in allowed regions and with no residues in generously or disallowed regions according to the Ramachandran plot (Fig. S4, Supplementary Materials). Furthermore, the model was also checked on the ProSA-web Server obtaining a Z-score of 6.14 which is within the range of scores typically found for native proteins of comparable size^35^ further confirming the model reliability. The last check was the comparison of the model with the one produced by the blind deep learning-based web resource trRosetta^36^: their structural alignment resulted in an RMSD lower than 2.6 Å, confirming the reliability of the entire procedure described above (Fig. S5, Supplementary materials).

The poly-purine RNA added via docking to the modelled protein to complete the HEV RNA Helicase complex was derived from the PDB structure 6JIM^37^, an RNA Helicase of the *Chikungunya virus*, which is a species belonging to the *Alsuviricetes* class like HEV and Tmv^38,39^.

### Docking ligands and analyzing complex stability

For the sake of identifying compounds with potential anti-viral properties, the model was targeted with natural bioactive compounds belonging to the cyclopenta[*b*]benzofuran/flavagline class to identify promising candidates to test in further dedicated investigations. This class of compounds has been described to have a broad spectrum of activity including insecticidal, antifungal, anti-inflammatory and anticancer activities to cite but a few^40^. However, the mechanisms of action underpinning those activities still need to be clarified, though RCG and other flavagline analogues proved to target prohibitins and to exert inhibitory activity towards RNA Helicases^27^. SLV (Fig. 1B) was also included in the study as it was an already known inhibitor of HEV replication, although the underpinning mechanisms and molecular targets still need clarifications^24–26^. Moreover, silvestrol aglycone (S–Ag;CID 24178739, Fig. 1B) was analysed due to the chemical similarity with both RCG and SLV, along with two virtual decoys to assess the procedural performances (ZINC ID ZINC8584442 and ZINC8387186; further details are reported in the "Docking" section).

The position to dock the ligands within the ligand binding site was defined based on the PDB structure 5ZC9 showing RCG engaged in a well-defined base–base stacking interaction (Fig. S2, Supplementary Materials).

As reported in Table 1, RCG showed the highest docking score followed by all its analogues. All of them clearly showed a stacking interaction except for RCG6 and the two decoys. Considering that the higher the score, the stronger the interaction, according to previous evidence^41,42^, this result suggested the preferred interaction, and possibly the higher inhibitory activity, of RCG compared to the other analogues. On the other hand, SLV obtained a higher docking score than virtual decoys but sensibly lower than both RCG and S–Ag. Of note, the inactive decoys served to validate the model. In particular, the lack of activity was imputable to their incapability to keep a stable stacking interaction resulting in an enzyme inhibition. Therefore, the fact that they both showed no stacking interactions corroborated the model reliability.

**Table 1:** Docking PLP score obtained running GOLD. The highest the score the more likely the docking pose is optimal.

Ligand	PLP score
RCG	256.075
RCG1	224.929
RCG2	246.042
RCG3	224.077
RCG4	231.621
RCG5	228.042
RCG6	169.175
SLV	184.600
S–Ag	236.039
ZINC8584442	178.539
ZINC8387186	150.527

Once obtained the docking pose for each ligand, 25 ns long MD simulations were run to investigate the evolution of the built complexes. Based both on the 5ZC9 crystal structure and other evidence from the literature claiming the RCG stabilization of the protein-RNA complex^43^, the following geometrical rules were associated to ligands theoretically able to interact with and inhibit HEV RNA Helicase. Specifically, they should: I) keep the stacking interaction with the RNA stably; and II) preserve the stability of the RNA–protein complex avoiding RNA detachment.

**Figure 2:**
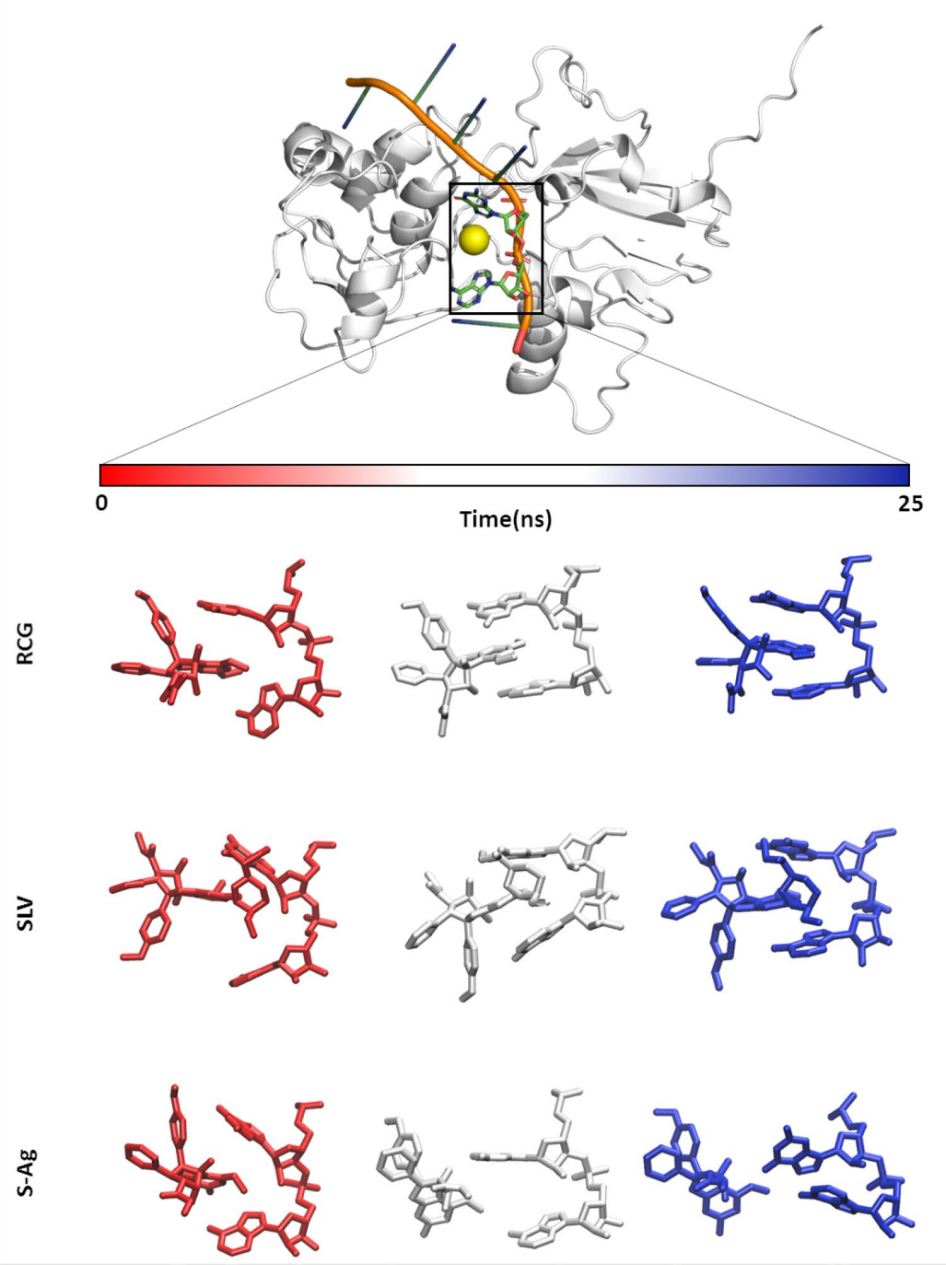
Protein-RNA-ligand complex and evolution over time of RCG, SLV and S–Ag with respect to the RNA bases. The protein is represented as a white cartoon, the RNA as an orange/green/blue cartoon while the two bases involved in the interaction with the ligand as green sticks. The yellow sphere represents the ligand docking-site. Under the time bar, starting from the top and moving to the bottom we can alternatively see the time-step sticks representation of both the RNA bases and RCG, SLV and S–Ag trajectories.

RCG and SLV were both engaged in a base–base stacking interaction, also stabilizing the protein-RNA complex with no appreciable RNA detachment (Fig. 2).

RCG1 did not show a proper stacking interaction and promoted the RNA detachment, pointing to its limited theoretical inhibitory activity against the viral RNA Helicase. Interestingly, this result is in line with the Pan et al. study reporting more than 500-fold activity decrease with respect to RCG against the human eIF4A1 ATP-dependent RNA helicase^27^. RCG2 caused a self-collapse of the RNA, promoting its detachment from the protein, and no stacking interactions were observed. This also suggested a low inhibitory potential, in line with the inactivity reported by Pan et al.^27^ against human RNA Helicase eIF4A1. RCG3, a less cytotoxic RCG analogue^27^, showed a single base interaction, rather than a base–base stacking interaction, and promoted the RNA detachment. Therefore, it was not deemed an efficient inhibitor. RCG4 and RCG6 both favoured the RNA detachment, with the latter reported as less active than the former, although RCG4 was found in an RCG-like stacking interaction with the RNA. They were not considered able to appreciably inhibit the HEV RNA Helicase activity.

S–Ag did not interact at all with the RNA. Shortly after the beginning of the MD simulation it slipped out the RNA chain and kept a likely unspecific surface interaction with a near protein portion (Fig. 2). This was probably due to the presence of the hydrophilic hydroxyl group in position R6 (Fig. 1), which prevented a proper a stacking interaction, which requires hydrophobic interactions, and to the absence of the SLV dioxane portion. Indeed, this part participates in the SLV stacking interaction confirming the Cencic and co-workers’ hypothesis claiming its crucial role for the SLV activity^44^. Concerning the two decoys, they both promoted RNA detaching, particularly ZINC8584442. This, along the lack of base–base stacking, was expected for inactive compounds and confirmed the procedural performances.

**Figure 3:**
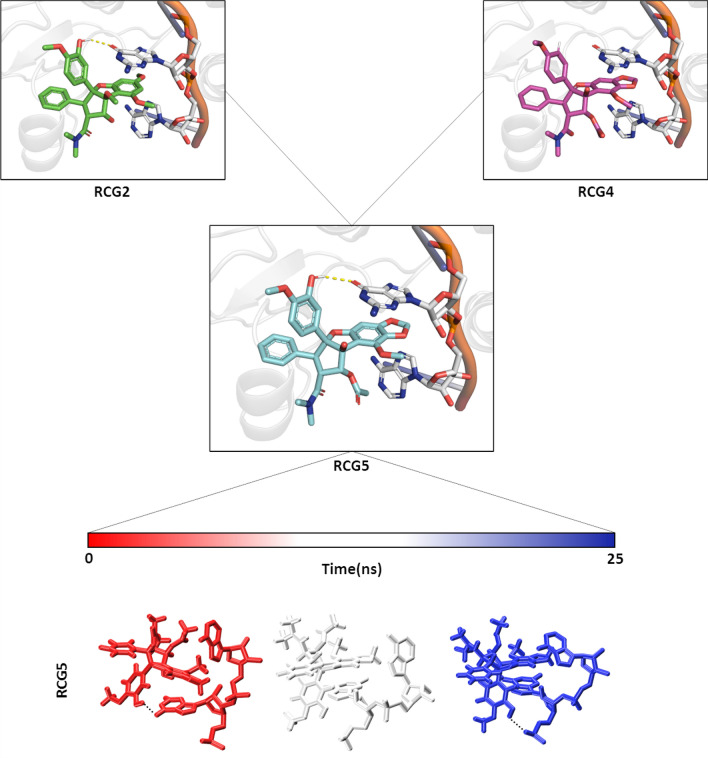
RCG2 is represented as green sticks, RCG4 as magenta sticks, RCG5 as pale-blue sticks. RNA bases involved in the interaction are represented as white sticks while the RNA backbone as orange cartoon. The yellow dashed lines represent the Hydrogen bond occurring between the R4 of both RCG5 and RCG2, and the RNA base. The time-step representation of the RCG5/RNA-bases trajectories is reported under the time bar. The black dashed lines represent both the starting Hydrogen bond and the final one.

Lastly, a novel RCG analogue, i.e. RCG5, was rationally designed and evaluated based on the calculated pose of other RCG analogues included in this study. Particularly, starting from docking poses it was noticed there was a hydrogen bond between the hydroxyl group in position R4 of RCG2 and an RNA base (Fig. 3). Moreover, looking at the progression of RCG2 MD simulation, the starting H-bond was not kept over time but a new one between the hydroxyl group in R4 and the RNA backbone was formed. This interaction was thought as favorably stabilizing the complex and theoretically able to enhance the activity of the compound. Therefore, in RCG5, the R4 of RCG4 (i.e., –H) was replaced with a hydroxyl group. RCG5 showed a stable stacking interaction while keeping the RNA–protein complex stable, steadily forming an H-bond with a phosphate group of the RNA backbone (Fig. 3). This newly designed compound needs further investigation and experimental evaluation to validate its HEV RNA Helicase inhibitory activity.

## Conclusion

Keeping in mind the intrinsic issues in running experimental trials on HEV related to its biology and to the shortage of a robust cell-culture system supporting its life cycle^45^, in silico approaches may ensure a useful framework of analysis to advance HEV understanding and design counteracting strategies. In this context, the present work provided a useful prioritization of compounds, most of which are not commercially available, supporting a knowledge-based and informed selection either for their synthesis or purification in future dedicated works. Overall, this study: (i) presented mechanistic insights on HEV RNA Helicases and its SLV/RCG-dependent inhibition; (ii) expanded the current understanding of the structure–activity relationship for SLV and RCG-related compounds; (iii) provided a blueprint for further analysis targeting HEV RNA Helicases. Moreover, this study described for the first time the inhibition of HEV RNA Helicase as a plausible mechanism of action of SLV, which was already described reducing HEV capability to penetrate cells. This ancillary mechanism, which deserves further investigation with high priority, was found plausible also for other RCG/SLV-like compounds and may complement the already reported activity of this HEV impairing agents on host RNA Helicases. Furthermore, there are evidence reporting SLV as well-tolerated in animals^40^ making it an interesting candidate as a feed additive. The described methodology, starting from the template selection moving to the actual modelling and testing, succeeded to build a reliable model able to qualitatively discriminate several ligands. In addition to this, the pipeline is highly versatile, flexible and it can be replicated on other HEV ORF1 domains, such as the RNA Dependent RNA Polymerase which has already been proved as a suitable target to inhibit virus replication^41^.

As a general remark, the high identity percentage among the HEV RNA Helicases of genotypes 1 to 4 (all above 88%; see Fig. S6, Supporting material), which are those available in the manually curated section of UniProt database (last accessed 3^rd^ August 2022), might suggest a broad inter-genotype activity for RCG, SLV and RCG6.

For the sake of data reproducibility and to support further studies, the 3D model of HEV RNA Helicases used in this work has been made publicly available (https://github.com/FC-MMLAB-UniPr/HEV3_Helicase_Model).

## Materials and methods

### Homology modelling

#### Sequence and template selection

The primary protein sequence used to model the 3D structure of HEV RNA Helicase was stored in the UniProt database (release 2021_04) with the Accession Code (AC) Q6J8G2^46^. As no crystallographic structures of HEV RNA Helicase were available in the Protein DataBank (PDB) at the time of analysis (last database access January 2022) a HEV RNA Helicase model was obtained via HM. T^47^To do so, a hybrid structure- and sequence-based strategy was developed as the search of publicly available PDB structures via the webserver BLAST interface (basic local alignment search tool, https://blast.ncbi.nlm.nih.gov/Blast.cgi)^43^, which is a goldstandard in this kind of study, did not gave useful structures for HM. Specifically, a set of non-eukaryotic proteins annotated as RNA Helicases (E.C 3.6.4.13, according to Brenda classification^32^) with available 3D structure in the Protein Data Bank was collected (228 entries at the time of analysis). To retrieve the 3D structure of this set of proteins, their UniProt ACs were downloaded and mapped towards the Protein Data Bank using the “Retrieve/ID Mapping” tool available on UniProt. Starting from their PDB IDs their 3D structures were iteratively downloaded(1940 at the time of analysis) in the .pdb format using a PyMol script developed in-house (available upon request). Each chain belonging to a .pdb file was converted to a fasta formatted file using an ad hoc script developed in-house (available upon request) and subsequentially concatenated in a unique *multi.fasta* file. This *multi.fasta* file was converted into a local database of sequences via the *makeblastdb* command set with default parameters (ncbi-blast+ suite; version 2.11.0 +)^47^ selecting the tabular output format. The best hit in terms of alignment score, e-value and identity percentage was considered to develop the homology model of HEV RNA Helicase.

#### Model generation and refinement

Modeller version 10.0 interfaced to Chimera (version 1.15)^48^ was used to generate the model of HEV RNA Helicase. The chosen template was the crystallographic structure with PDB code 3WRY (only the residues aligning the RNA Helicase domain were considered). Fifty models were generated, setting the inclusion of non-water heteroatoms, using a thorough optimization and choosing the best scored model according to zDOPE for subsequent analysis.

After computing a tentative model and checking its Ramachandran Plot with PROCHECK v3.5^49^, regions with improper dihedrals (namely, residues 30–33; 101–103, 111–113 and 97–99) were stepwise refined with the Modeller loop-refinement tool (version 10.0 interfaced to Chimera^50^ version 1.15) using the DOPE modelling protocol, generating 5 models and carrying forth to the analysis the best model according to the zDOPE scoring.

Before using the model, a final assessment was performed by re-building its Ramachandran Plot and checking its Z-score on the ProSa-web Server^35^ to verify the proper topology.

#### Building the model-RNA complex

The HEV RNA Helicase-RNA complex was obtained docking the RNA sequence to the previously built RNA Helicase model via ClusPro 2.0^38^ set with default parameters. The input RNA structure was chosen based on the following protocol. First, all the structures of RNA Helicase containing RNA were download from Protein Data Bank and aligned to the model previously obtained. The RNA of the structure with the most similar organization (i.e., with the lowest RMSD value calculated in PyMol using the super command) was chosen (PDB ID 6JIM). In the last step, the RNA of 6JIM and the HEV RNA Helicase model were uploaded on ClusPro 2.0^38^. Out of the obtained complexes the analysis of one of the mostly hydrophobic-favoured poses, showing the RNA molecules arranged in the same area as 6JIM structure, was carried forth. The sequence was than edited to a poly-purine fragment in agreement with previous studies reporting its suitability to interact with SLV-related compounds^51^.

### Docking

Docking studies were performed with GOLD (Genetic Optimization for Ligand Docking; version 2021.3)^52^ to provide a plausible binding architecture for a set of flavagline compounds. The 3D structure of RCG (Fig. 1A), SLV (Fig. 1B) and S–Ag (Fig. 1B) were downloaded from PubChem (https://pubchem.ncbi.nlm.nih.gov/; CID 331783, CID 11787114 and CID 24178739, respectively)^53^ in the .sdf format. The other RCG analogues (Fig. 1A) were generated editing the RCG structure using the PyMol Builder tool (version 2.3.0) and further optimized using Chimera (version 1.15)^50^ with the Minimize Structure tool (5000 steepest descent steps and 100 conjugate gradient steps).

The RNA Helicase model was used as input structure and the space to arrange ligands was set based on the architecture of binding of the RCG in the human eIF4A1 ATP-dependent RNA helicase having PDB code 5CZ9. The structure was visually aligned to the HEV RNA Helicase model, and the binding site was defined in a 10 Å-radius sphere around the centroid of the inter-bases space occupied by RCG in 5CZ9 structure (Fig. S1, Supplementary Materials). RCG was docked first generating 100 poses with no positioning constraints, setting the ligand fully flexible and allowing polar protein hydrogens free to rotate. The best scored pose according to PLP Scoring function (256 units; the higher the score, the more probable and favoured the ligand interaction) showed a comparable binding architecture to the crystallographic binding pose of RCG. Such pose was then used as a position restraint setting the similarity option with shape overlap (weight constraint 200). This helped docking SLV and other analogues facilitating their proper arrangement into the binding site.

Virtual decoys were also generated to test procedure performances via the DUD-E database Generate tool (http://dude.docking.org/)^54^. The 50 decoys generated were ranked according to chemical similarities to SLV using LiSiCA algorithm^55^ and the two extremes (the most similar and the most dissimilar compound; ZINC ID ZINC8584442 and ZINC8387186, respectively) (Fig. S2 Supplementary materials) were docked for the sake of procedure validation (see in the “Docking ligands and analyzing complex stability” section for further details).

### Molecular dynamics

Molecular dynamics (MD) simulations were performed to investigate the overall geometrical stability of HEV RNA Helicase-ligands complexes over time. The adopted software was GROMACS (version 2019.4)^56^ with CHARMM27 all-atom force field parameters support^57^. All the ligands have been processed and parameterized with CHARMM27 all-atom force field using the SwissParam tool (http://www.swissparam.ch)^58^. Input structures were solvated with SPC/E waters in a rhombic dodecahedron periodic boundary condition, and counter ions (Na^+^ or Cl^−^) were added to neutralize the system. Prior to perform molecular dynamic simulations, the systems were energetically minimized to avoid steric clashes and to correct improper geometries using the steepest descent algorithm with a maximum of 50,000 steps. Afterwards, all the systems underwent isothermal (300 K, coupling time 0.1 ps) and isobaric (1 bar, coupling time 2 ps) 100 ps simulations before running a 25 ns MD simulation.

### Multiple sequence alignment

A multiple sequence alignment has been performed to infer the activity of RCG, SLV and RCG6 over the HEV RNA Helicases of different HEV genotypes. The analysis focused on genotypes 1 to 4 as they were those publicly available on the manually curated and reviewed section of UniProt database (last accessed 3^rd^ August 2022). We performed the analysis using Clustal Omega Web Server (https://www.ebi.ac.uk/Tools/msa/clustalo/) with default parameters^59^.

## Supplementary Information

Supplementary Information.

## Data Availability

Data are available upon a formal request to the corresponding author. The HEV RNA Helicase model used in this study is available at https://github.com/FC-MMLAB-UniPr/HEV3_Helicase_Model.
