# Supplementary material for: In silico study on the Hepatitis E virus RNA Helicase and its inhibition by silvestrol, rocaglamide and other flavagline compounds
Source: Sci Rep. 2022 Sep 15;12:15512. doi: 10.1038/s41598-022-19818-w (PMC9477874; doi:10.1038/s41598-022-19818-w)
Supplement: Supplementary file 1 — Supplementary Information. [file 41598_2022_19818_MOESM1_ESM.docx]

**Supporting Material**

***In silico* study on the Hepatitis E Virus RNA Helicase and its inhibition by silvestrol, rocaglamide and other flavagline compounds**

Lorenzo Pedroni ^1, #^, Luca Dellafiora ^1, #,*^, Maria Olga Varrà ^1^, Gianni Galaverna ^1^, Sergio Ghidini ^1,*^

^1^ Department of Food and Drug, University of Parma, 43124 Parma, Italy

^#^ These authors contributed equally to the work

^*^ Corresponding Authors: Luca Dellafiora, Department of Food and Drug, University of Parma, 43124 Parma, Italy; Phone: +39 0521 906079, Email: luca.dellafiora@unipr.it. Sergio Ghidini, Department of Food and Drug, University of Parma, 43124 Parma, Italy; Phone: +39 0521 902761, Email: sergio.ghidini@unipr.it


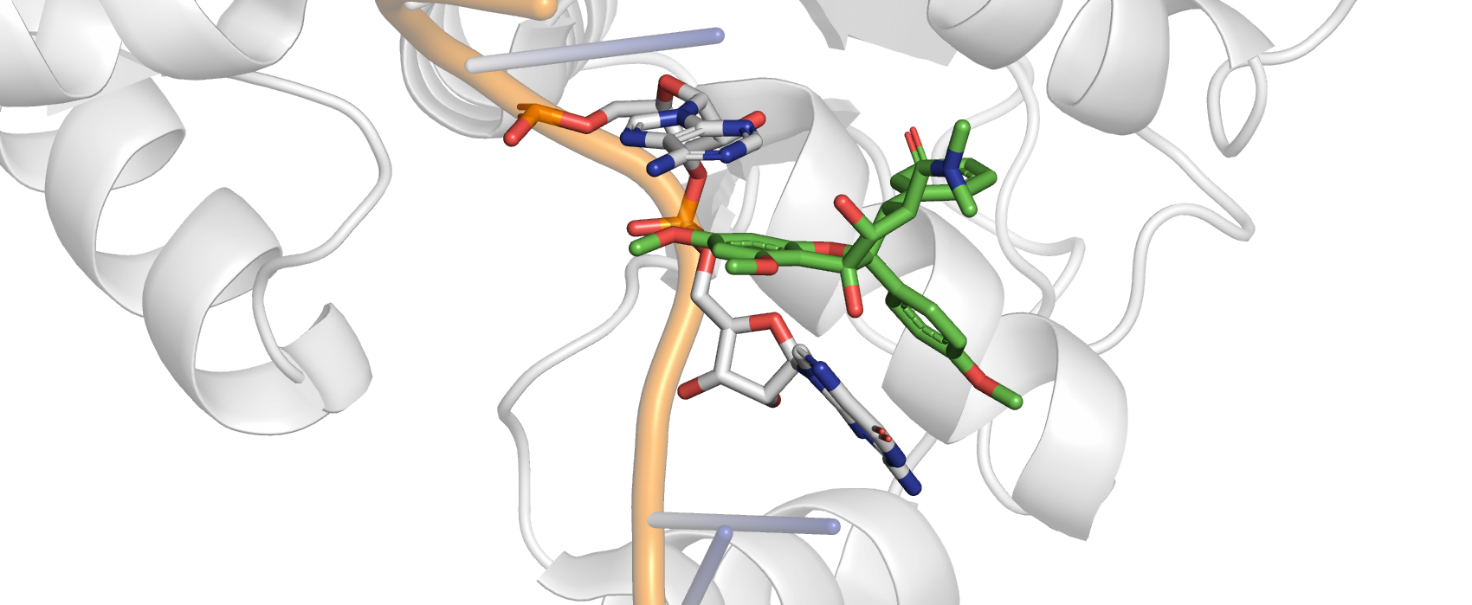


**Figure S1.** RCG pose within the PDB 3D structure with code 5ZC9. The RCG is represented as green sticks while the RNA bases are in white sticks.


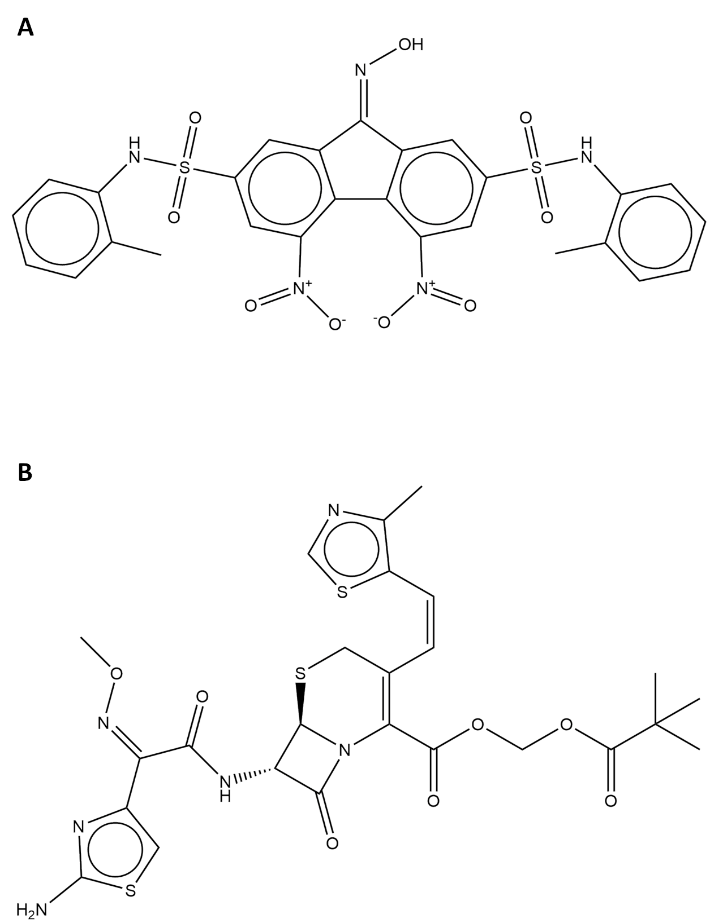


**Figure S2.** Structures of the virtual decoys used in this study. **A**. ZINC8387186 **B**. ZINC8584442


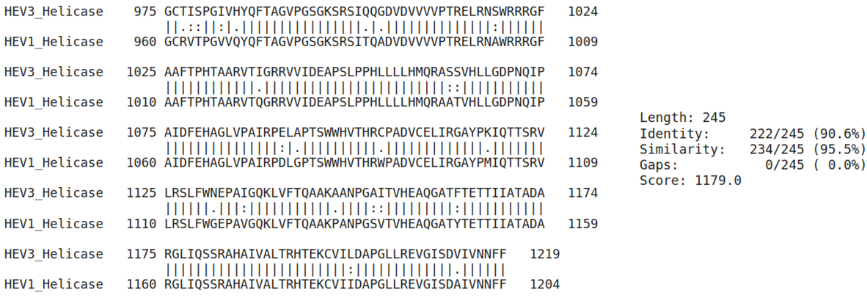


**Figure S3.** Sequence alignment of the HEV1 RNA Helicase with the HEV3 RNA Helicase.


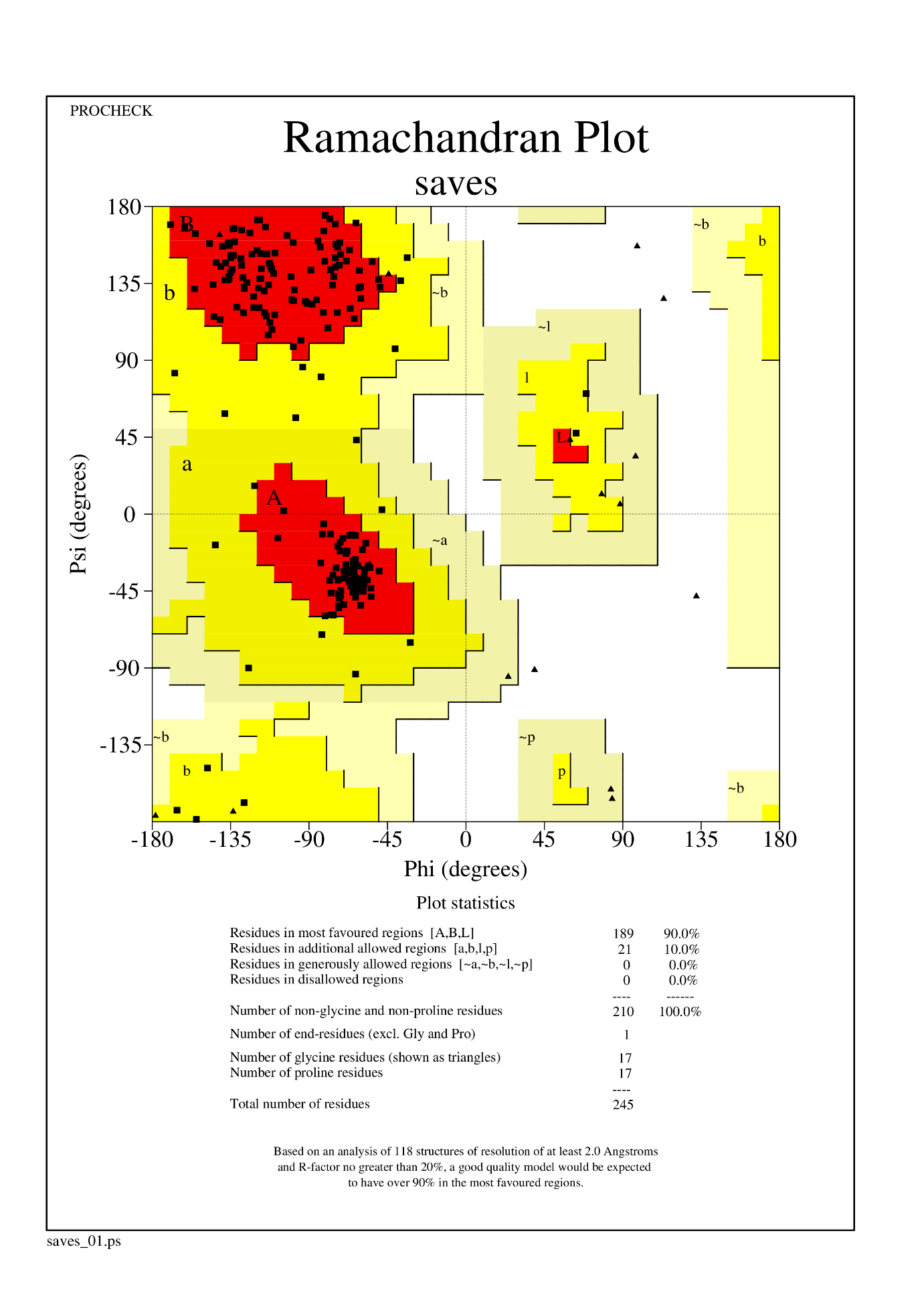


**Figure S4.** Ramachandran Plot of the HEV RNA Helicase model computed via PROCHECK v3.5.


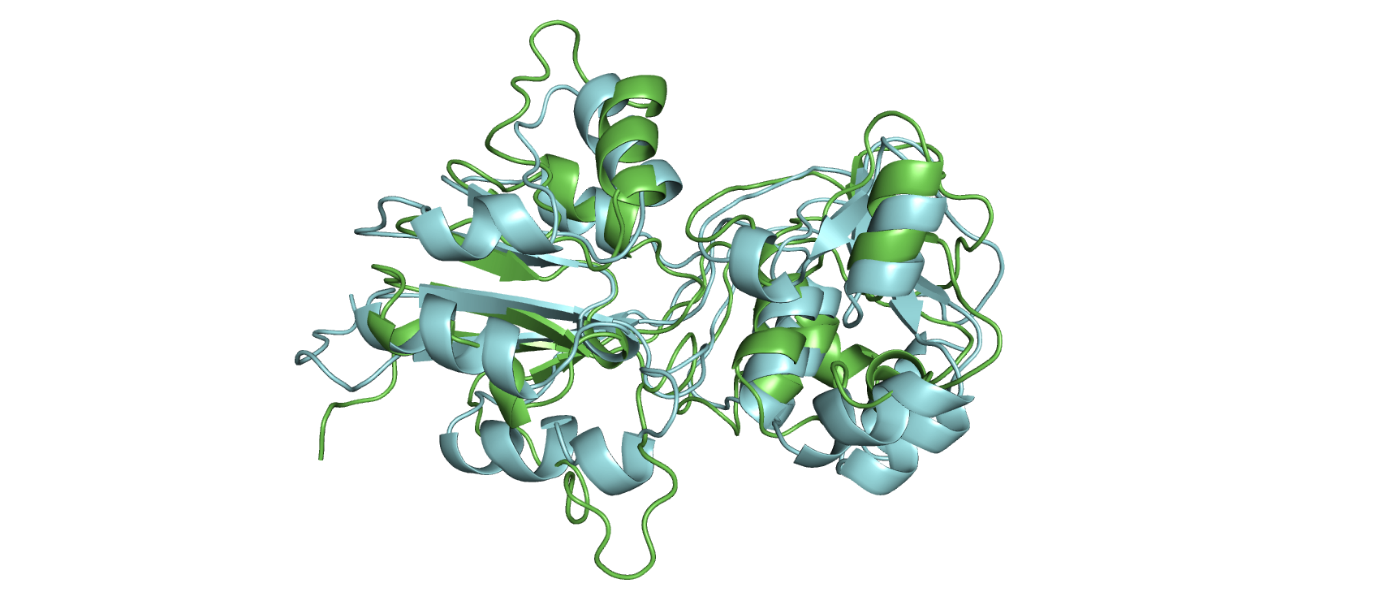


**Figure S5.** Structural alignment of the HEV RNA Helicase model (green cartoon) with the model built via the trRosetta web-server (pale-blue cartoon).


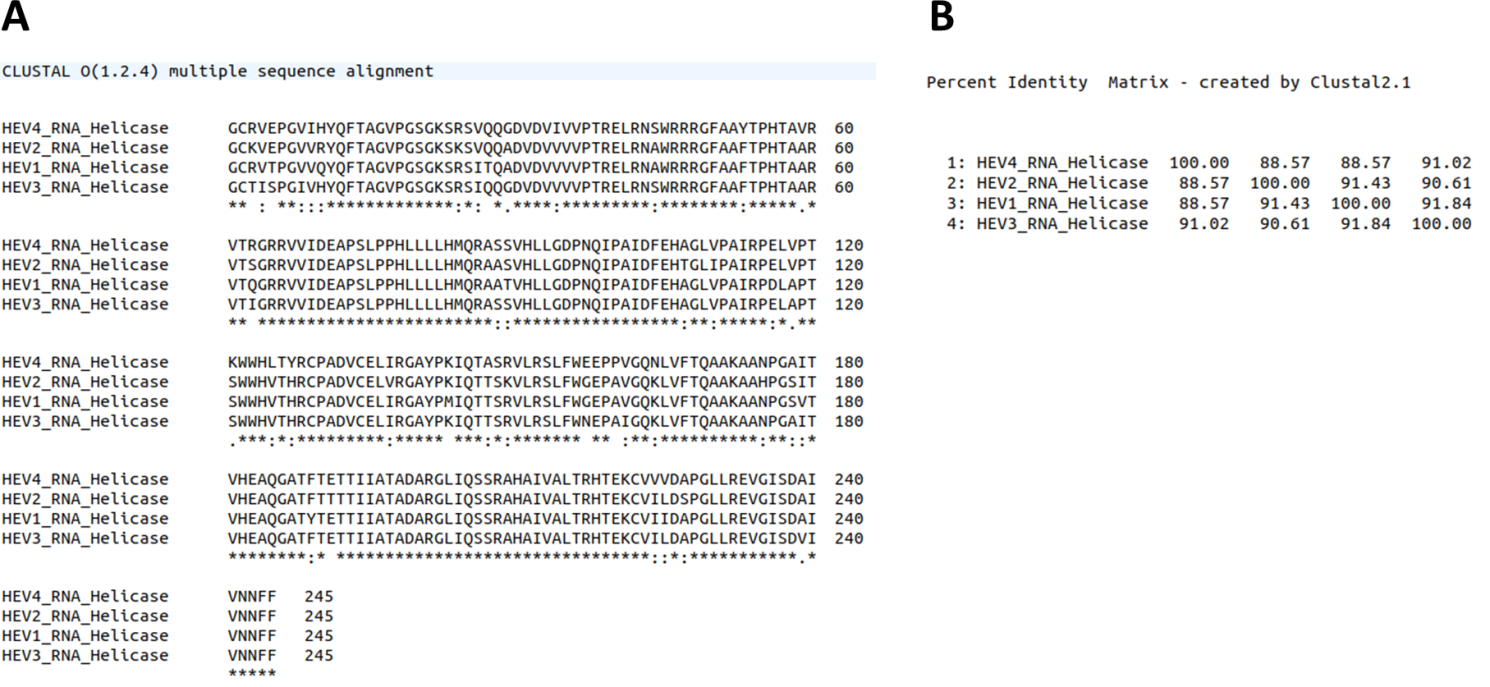


**Figure S6**. Multiple sequence alignment of HEV RNA Helicases from genotypes 1 to 4 (**A**) and related identity percentage matrix (**B**). The asterisk indicates residue identity, while column (:) indicates conservation between groups of strong similar properties, and dot (.) indicates conservation between groups of weakly similar properties. HEV1, HEV2, HEV3 and HEV4 refers to the RNA Helicases of HEV genotype 1, 2, 3 or 4, respectively.
